# Effect of acupuncture on chronic bronchitis: A protocol for systematic review and meta-analysis

**DOI:** 10.1097/MD.0000000000020676

**Published:** 2020-06-12

**Authors:** Dongdong Mao, Yanli Deng, Leixiao Zhang, Ying Zhao, Ying Li, Fei Wang

**Affiliations:** a Hospital of Chengdu University of Traditional Chinese Medicine/Clinical Medical College of Chengdu University of Traditional Chinese Medicine; b Sichuan Second Chinese Medicine Hospital; c Acupuncture and Tuina School, Chengdu University of Traditional Chinese Medicine, Chengdu, Sichuan, China.

**Keywords:** acupuncture, chronic bronchitis, protocol, systematic review

## Abstract

**Introduction:**

Chronic bronchitis (CB) is a clinically common and recurrent respiratory disease. However, many trials have shown that acupuncture can effectively treat CB. There is currently no systematic review of this therapy. The plan is to evaluate the effectiveness and safety of this treatment in patients with CB.

**Methods and analysis:**

This systematic evaluation will entail an electronic and manual search of all acupuncture for CB from inception to December 31, 2020, regardless of the publication status or language. Databases include PubMed, Embase, Springer, Web of Science, the Cochrane Library, the World Health Organization International Clinical Trial Registration Platform, the Chinese Medicine Database, the China National Knowledge Infrastructure, the Chinese Biomedical Literature Database, the China Science Journal Database, and the Wanfang Database. Other sources of information, including bibliographies and meeting minutes for identified publications, will also be searched. A manual search for grey literature, including unpublished conference articles will be performed. Additionally, any clinical randomized controlled trials related to acupuncture for CB, regardless of the publication status and language limitations, will be included in the study. Study selection, data extraction, and research quality assessments will be conducted independently by 2 researchers. The main result was the Change in cystic fibrosis transmembrane conductance regulator function as measured by sweat chloride analysis or treatment effect. Secondary outcomes included Quality of life (eg, SF-36), change in Breathlessness, Cough, and Sputum Scale score, follow-up relapse rate, and adverse events. The system searches for randomized controlled trials of this therapy for CB. Implement the Cochrane RevMan V5.3 bias assessment tool to assess bias risk, data integration risk, meta-analysis risk, and subgroup analysis risk (if conditions are met). Mean difference, standard mean deviation, and binary data will be used to represent continuous results.

**Results:**

This study will provide a comprehensive review and evaluation of the available evidence for the treatment of CB using this therapy.

**Conclusion:**

This study will provide new evidence to evaluate the effectiveness and side effects of acupuncture on CB. Because the data are not personalized, no formal ethical approval is required.

**PROSPERO registration number:**

CRD42020170287

## 1 Introduction

### 1.1 Description of the condition

Chronic bronchitis (CB), defined by chronic cough and sputum, affected 5% of US adults aged 45 years or older in 2018.^[1]^ Presence of CB is an indication for pulmonary function testing; however, CB is not included in the diagnostic criteria for chronic obstructive pulmonary disease (COPD) and frequently occurs without comorbid COPD.^[2–4]^ CB is characterized by chronic cough and mucus secretion from airways for at least 3 months in each of 2 successive years where no specific cause can be identified.^[5]^ CB was considered an important step in COPD development. Although CB is no longer considered as an early stage of COPD, its presence definitely remains a marker of increased risk of developing COPD, and its symptoms are often underestimated.^[6]^

A recent cohort study involving nearly 9000 patients also showed that CB was common in patients with COPD.^[7]^ A survey in China concluded that about 85% of the pulmonary heart disease morbidity is secondary to CB and emphysema.^[8]^ CB affects all race/ethnic groups in the United States, with prevalence rates being highest among non-Hispanic whites and blacks (47.3/1000 and 48.6/1000, respectively). Approximately 942,000 Hispanics/Latinos in the United States are affected by CB (prevalence rate 28.8/1000).^[9]^ Although the most established risk factor for CB is active smoking, several studies have shown CB to be present in 22% to 37% of never-smokers.^[10]^ Nonobstructive CB was associated with adverse respiratory health outcomes, particularly in ever smokers.^[11]^

The prevalence of CB was higher among Dominicans than other hispanic/latino heritages. CB was more prevalent among US-born participants and those exposed to cleaning and disinfecting solutions.^[12]^ Occupational exposure to dust, gases, and fumes, as well as pesticides, increased the risk of respiratory symptoms and CB.^[13]^ CB, asthma, and rhinitis were associated with cardiovascular risk factors and diseases. In particular, CB shared with cardiovascular diseases almost all risk factors and was strongly associated with a higher risk of heart disorders and intermittent claudication.^[14]^ The unhealthy lifestyles (smoking, high alcohol consumption, and sedentariness), hypertension, and dyslipidemia may predict a greater risk of CB.^[14]^

Cough is one of the most common reasons patients seek medical attention.^[15]^ CB symptoms should be monitored intensively to allow early detection and prevention of COPD. In addition to COPD risk, CB may also increase mortality risk. In the UK, it results in 5% of death. When acute exacerbations occur, the mortality is even larger.^[16]^ The frequent exacerbations of CB increase morbidity and mortality worldwide, leading to recurrent hospitalizations, pulmonary function impairment, and chronic airway limitation.^[17]^

Although antibiotics and expectorants have obvious effects on CB, the most common side effects are typical rashes and gastrointestinal upset. As a green physical alternative therapy, acupuncture has been recognized for its effectiveness in respiratory diseases.^[18,19]^ Therefore, in this article, we will determine the effectiveness and safety of acupuncture in the treatment of CB by comparing acupuncture with various types of control interventions.

### 1.2 Description of the intervention

Acupuncture has been proved to be efficient in China for >2000 years and it had been widely used in treatment of varieties of diseases. Acupuncture is based on Traditional Chinese Medical (TCM) theory in which health is deemed as keeping balance of energy that is called Qi flowing a circulation through the channels and meridians in the body. Acupuncture is one of the key components of TCM therapies in which thin needles are inserted into the body. Acupuncture corrects the imbalance of the flowing of Qi via a needle inserted into special acupoints along the meridians.^[20]^ Acupuncture can not only improve the clinical symptoms and signs of patients with CB, improve the quality of life, but also improve their immune function. Studies have confirmed that acupuncture can reduce the inflammatory damage of bronchial mucosa by reducing the expression levels of serum interleukin (IL)-8 and tumor necrosis factor-α; at the same time, it can increase serum IL-2 levels, enhance the body's ability to fight inflammatory response, and improve immune function.^[21]^

### 1.3 Objectives

To develop treatment recommendations, we systematically evaluated the efficacy and safety of acupuncture for CB.

## 2 Methods

### 2.1 Study registration

PROSPERO registration number is CRD42020170287. This protocol report is structured according to the Preferred Reporting Items for Systematic Reviews and Meta-Analyses Protocols (PRISMA-P) statement guidelines.^[22]^ The review will be conducted in accordance with the PRISMA guidelines and follows the recommendations of the Cochrane Handbook for Systematic Reviews of Interventions.^[23,24]^ If we refine the procedures described in this protocol, we will update the record in the PROSPERO and disclose them in future publications related to this study.

### 2.2 Inclusion criteria for study selection

#### 2.2.1 Types of study

To evaluate the efficacy of acupuncture in the treatment of CB, this article only reviewed the randomized controlled trial (RCT) between acupuncture and the control group. In addition, both Chinese and English publications are subject to language restrictions. All RCTs that are not subject to publication state constraints will be included. If the experiment shows that the phrase is random and the blind method is not restricted, it will be regarded as a random study. Animal mechanism studies, case reports, self-controlled, non-RCTs, random crossover studies, and quasi-randomized trials will be excluded.

#### 2.2.2 Types of participants

Regardless of sex, age, ethnicity, education, and economic status, patients with CB who meet the following diagnostic criteria (as diagnosed using any recognized diagnostic criteria) will be included.

#### 2.2.3 Types of intervention

Acupuncture refers to a method of stimulating acupuncture points, including hand needles, leather needles, plum blossom needles, ear needles, electroacupuncture, or fire needles. Other methods such as acupressure, moxibustion, laser acupuncture, drug acupuncture, dry needle, or transcutaneous electrical nerve stimulation will be excluded. Sham acupuncture includes acupuncture at inappropriate acupoints, sham acupuncture at acupoints, sham acupuncture at nonacupoints, nonpiercing acupuncture, and sham intervention.

1. Acupuncture compared with no treatment
2. Acupuncture compared with placebo or sham treatment
3. Acupuncture compared with other active therapies
4. Acupuncture in addition to active therapy compared with the same active therapy.

We will assess and compare the AT according to how the acupuncturists have been trained and educated, on their clinical experience, on total numbers of acupuncture sessions, and on the treatment duration and frequency, etc.

#### 2.2.4 Types of outcome measures

The primary outcome was the Change in cystic fibrosis transmembrane conductance regulator function as measured by sweat chloride analysis or treatment effect. Secondary outcomes included Quality of life (eg, SF-36), Change in Breathlessness, Cough, and Sputum Scale score, follow-up relapse rate, and adverse events. The system review will be performed independently.

### 2.3 Data sources

Our systematic review will search all RCTs for acupuncture on CB, electronically and manually, regardless of publication status and language, by December 31, 2020. Databases include: PubMed, Embase, Springer, Web of Science, Cochrane Library, WHO International Clinical Trials Registry Platform, Traditional Chinese Medicine databases, China National Knowledge Infrastructure, China Biomedical Literature Database, Chinese Scientific Journal Database (VIP), and Wan-Fang database. Other sources, including reference lists of identified publications and meeting minutes, will also be searched. Manually search for grey literature, including unpublished conference articles.

### 2.4 Search strategy

The search strategy will be followed the PRISMA guidelines. The key search terms are (“chronic bronchitis” OR “bronchitis”) AND (“acupuncture” OR “hand needles” OR “leather needles”) AND (“randomized”). The search strategy will be adapted to different databases demands. Search strategy in PubMed is shown in Table 1.

**Table 1:**
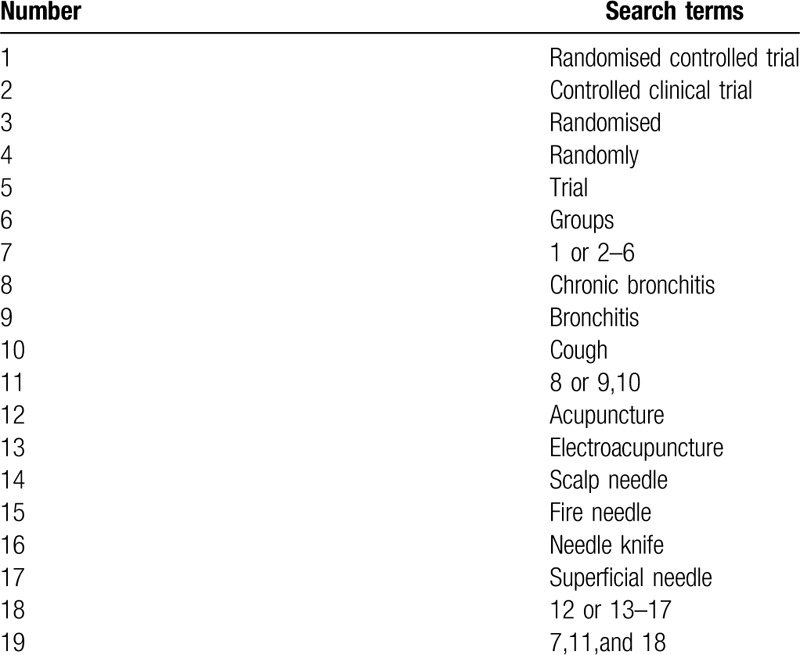
PubMed search strategy.

### 2.5 Data collection and analysis

#### 2.5.1 Selection of studies

**Figure 1:**
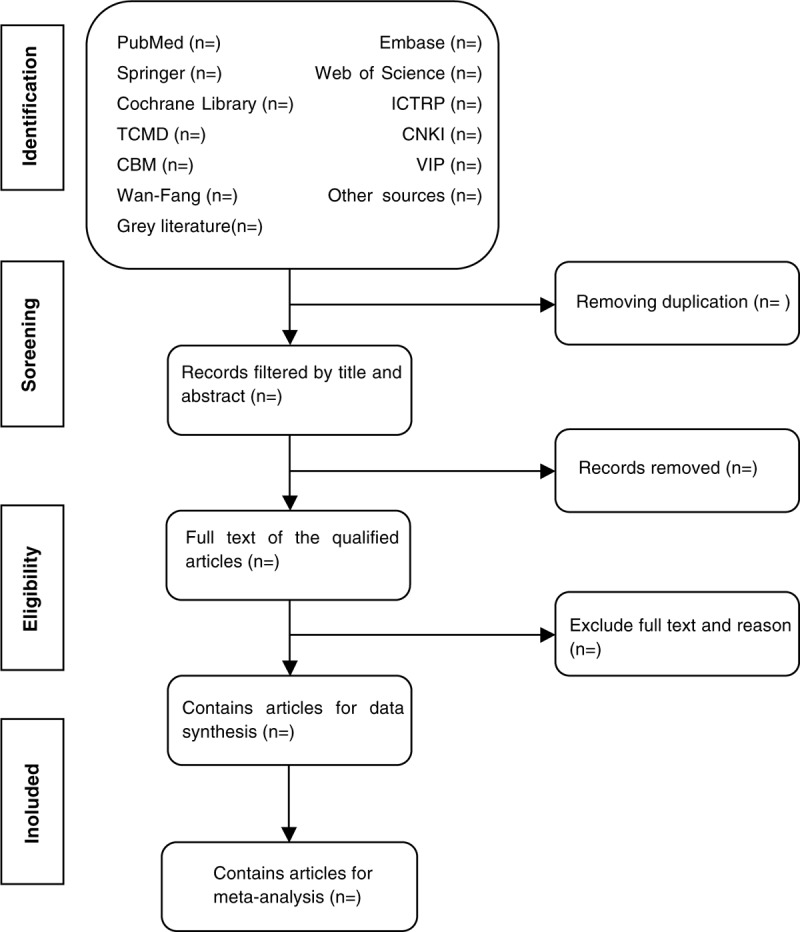
Flow diagram of studies identified.

Before literature retrieval, all reviewers are trained to ensure a basic understanding of the background and purpose of the review. In the literature screening process, we will use EndNote software (V.X8) document management software. The 2 comment author (DDM and YLD) will be in strict accordance with the inclusion criteria, independent screen all retrieval research, read the title, abstract and key words in the literature, and determine which meet the inclusion criteria. We will obtain the full text of all relevant studies for further evaluation. Excluded studies will be documented and explained. If there is a disagreement in the selection process, it will be discussed by the 2 authors (DDM and YLD) and the third author (FW) and arbitrated if necessary. If necessary, we will contact the trial author for clarification. The primary selection process is shown in a PRISMA flow chart (Fig. 1).

#### 2.5.2 Data extraction and management

The authors will extract data independently from the selected report or study and fill out the data extraction form. We will obtain data on general information, participants, methods, interventions, outcomes, results, adverse events, conflicts of interest, ethical recognition, and other information. For publications with insufficient or ambiguous data, we will attempt to obtain information from the corresponding authors by e-mail or telephone. Any differences will be resolved through discussions between the 2 authors, and further differences will be arbitrated by the third author (FW).

#### 2.5.3 Assessment of risk of bias and reporting of study quality

The authors (DDM and YLD) will use the Cochrane Collaboration's bias risk assessment tool to assess the risk of bias in all included studies. We will assess the risk of bias in the following areas: sequence generation, assignment sequence hiding, the blindness of participants and staff, and result evaluators, incomplete outcome data, selective outcome reporting, and other sources of bias. This review uses L, U, and H as the key to these assessments, where L (low) indicates a lower risk of bias, U (unclear) indicates that the risk of bias is uncertain, and H (high) indicates a higher risk of bias. If inconsistent results appear, the final decisions will be made by the third author (FW). Information on the risk of biased assessments included in the study is summarized in tabular form and the results and impacts are critically discussed. If the information is ambiguous, we will try to contact the author. For repeated publications, we only select the original text.

#### 2.5.4 Measures of treatment effect

Data analysis and quantitative data synthesis will be performed using RevMan V.5.3. For continuous data, if there is no heterogeneity, we will use mean difference (MD) or standard MD to measure the therapeutic effect of 95% confidence interval (CI). If significant heterogeneity is found, a random-effects model will be used. For dichotomous data, we will use the 95% CI risk ratio (RR) for analysis.

#### 2.5.5 Unit of analysis issues

We will include data from parallel group design studies for meta-analysis. Only the first phase of the data will be included in the random crossover trial. In these trials, participants were randomly divided into 2 intervention groups and individual measurements for each outcome of each participant were collected and analyzed.

#### 2.5.6 Management of missing data

If the primary result has missing or incomplete data, we will contact the author of the communication to obtain the missing data. If it is never available, exclude the experiment from the analysis.

#### 2.5.7 Assessment of heterogeneity

We will use the Review Manager to assess efficacy and publication bias (version 5.3, Nordic Cochrane Centre, Copenhagen, Denmark). The forest map is used to illustrate the relative strength of the effect. The funnel plot is used to illustrate the bias because the number of trials exceeds 10. If a significant difference is detected, a random-effects model will be used.

#### 2.5.8 Assessment of reporting biases

We will use a funnel plot to detect report bias. If >10 trials are included, the funnel plot will be used to assess the reported bias. If the funnel plot is found to be asymmetrical, analyze the cause using Egger method. We will include all eligible trials regardless of the quality of the method.

#### 2.5.9 Data synthesis

We will use RevMan for all statistical analysis. If considerable heterogeneity is observed, a 95% CI random effects model will be used to analyze the combined effect estimates. Subgroup analysis will be performed with careful consideration of each subgroup if necessary.

#### 2.5.10 Subgroup analysis

There is no presubgroup plan. Subgroup analysis was performed based on control interventions and different outcomes.

#### 2.5.11 Sensitivity analysis

Based on sample size, heterogeneity quality, and statistical models (random or fixed-effect models), we will perform sensitivity analysis.

#### 2.5.12 Grading the quality of evidence

The quality of evidence for all outcomes will be judged by the Grading of Recommendations Assessment, Development, and Evaluation (GRADE) working group approach. Bias risk, consistency, directness, precision, publication bias, are aspects of our assessment. High, medium, low, or very low represent the 4 levels of evaluation.

## 3 Discussion

CB is a major refractory and recurrent public health problem. About 3.4% to 22% of people worldwide suffer from CB.^[9]^ It is associated with a variety of clinical consequences, including accelerated lung function decline, increased risk of deterioration, reduced health-related quality of life, and may increase mortality. Acupuncture has been used in China to treat a vast array of pulmonary diseases from asthma to CB, from tuberculosis to viral pneumonia, and so on.^[19]^

The evaluation of this systematic review will be divided into 4 parts: identification, inclusion of literature, data extraction and comprehensive data analysis. According to the Cochrane method, this study is based on the analysis of clinical RCT evidence at home and abroad, and conducts search and screening of evidence-based medical evidence in major electronic literature databases to provide clinicians with more convincing evidence in decision-making and better guide clinical treatment.

## Author contributions

**Conceptualization:** D. Mao.

**Methodology:** Y. Deng.

**Software:** L. Zhang.

**Supervision:** Y. Zhao.

**Validation:** Y. Li, F. Wang.

**Writing – original draft:** D. Mao, Y. Deng, L. Zhang, Y. Zhao.
